# General and reproductive health outcomes among female greenhouse workers: a comparative study

**DOI:** 10.1186/s12905-020-00966-y

**Published:** 2020-05-13

**Authors:** Tahereh Rahimi, Foozieh Rafati, Hamid Sharifi, Fatemeh Seyedi

**Affiliations:** 1 Department of Public Health, Faculty of Health, Jiroft University of Medical Sciences, Jiroft, Iran; 2 Department of Nursing, Faculty of Nursing and Midwifery, Jiroft University of Medical Sciences, Jiroft, Iran; 3 HIV/STI Surveillance Research Center, and WHO Collaborating Center for HIV Surveillance, Institute for Futures Studies in Health, Kerman University of Medical Sciences, Kerman, Iran; 4 Department of Anatomical Sciences, Faculty of Medicine, Jiroft University of Medical Sciences, Jiroft, Iran

**Keywords:** Female greenhouse worker, Reproductive age, Pregnancy outcome, Iran

## Abstract

**Background:**

Agricultural activities as well as exposure to pesticides could have many adverse effects on health status and reproductive outcomes especially in reproductive aged greenhouse workers. The present study aimed to compare the general and reproductive health outcomes between female greenhouse workers and housewives.

**Methods:**

This cross-sectional study was conducted on 645 females of reproductive age. They were categorized into two groups according to occupation in greenhouse (308 female greenhouse workers as exposed group and 337 housewives as control group). Participants *were interviewed using a questionnaire* about reproductive outcomes and occupational status. Clinical measures include blood pressure (BP), pulse and respiratory rate (PR and RR), body mass index (BMI) and hematological parameters. Mann-Whitney U test was used to assess differences between two groups in quantitative variables. Chi-square or Fisher’s exact tests was used to determine the differences in the distribution of categorical variables. Data was analyzed using SPSS software version 16.

**Results:**

The average daily working hours of the greenhouse workers were 6.94 ± 1.99 h. Only 44.3% of them used personal protective equipment. Data revealed that the rate of spontaneous abortion, infertility, low birth weight (LBW), abnormal births and preterm birth were significantly higher among the greenhouse workers compared to the control group (*p* ≤ 0.05). The average number of female infants in greenhouse workers was significantly higher than the control group (*p* ≤ 0.05). Clinical measurements indicated an increase in RR of greenhouse workers and mean of BMI was decreased in them. Hematological parameters demonstrated that there was a significant increase in white blood cells (WBC) and significant decrease in hemoglobin (Hb), hematocrit (HCT), mean corpuscular volume (MCV) and mean corpuscular hemoglobin concentration (MCHC) among the female greenhouse workers compared to the control (*p* ≤ 0.05).

**Conclusion:**

Local government efforts is needed to address associated issues including acute effects on health and long-term health risks, resulting from pesticide exposure to greenhouse workers, and gender differences should be considered. Also, occupational health and safety training is necessary and can be helpful in reducing adverse reproductive outcomes.

## Background

In many developing countries, women are responsible for a large part of the agricultural production process [1, 2] demonstrating the importance of potential health threats in their workplace. In general, farmers are faced with a wide range of environmental and physical harmful factors such as climatic conditions, heat, dust, noise and vibration [3–5]; psychological factors such as high stress and depression [6]; biological factors such as exposure to bacteria, viruses, fungi and parasites [7] and chemical factors such as exposure to pesticides [8].

*Exposure to pesticides* has many adverse effects and is a concern for human public health especially on female health status. The scientific evidence indicated that respiratory infections, hypertension, gynecological diseases, rheumatoid and joint diseases, pregnancy complications, skin infection, asthma, and diabetes mellitus are common among female farmers [9, 10]. In addition, female engaged in *agriculture* are more susceptible to experience reproductive disorders including menstrual disturbances, reduced fertility, preterm or postterm birth, spontaneous abortion, stillbirth, congenital abnormalities and LBW due to pesticide exposure [11].

Several investigations have shown that pesticide exposure lead to adverse pregnancy outcomes. For example a study on California’s agricultural regions indicated women who exposed to some agricultural pesticides interfere with increased risk of preterm birth in the first and second trimester of pregnancy approximately 3–7% [12]. Another study on Egyptian women revealed that pregnancy-related problems including abortion and preterm birth are more common among women who farm. Moreover, the increased estradiol and progesterone levels, as well as liver enzymes and a decreased FSH level were significantly observed in female farmers [13].

The study on Bolivian farmers manifested that women reported more symptoms of acute illness including headache, burning eyes, redness of the skin and chills during or after exposure to pesticides. Also, female exposed to pesticide during pregnancy or lactation experienced approximately 50% of abortions and 17% of abnormal births [14].

A greenhouse is a non-open space that provides a suitable environment for the growth of plants; despite outside weather conditions. Atmospheric carbon dioxide levels, temperature, light intensity and humidity can be handled in order to encourage *plant’s growth* [15]*.* Because of high temperature, humidity and other physical characteristics of the greenhouse environment, exposure to pesticide and absorption increase in greenhouse compared to open-air agriculture [16].

Kerman province has been located in the south of Iran. There are a large number of greenhouses in the south of this province estimating more than two thousand hectares in 2019. This area is the first place ranked for greenhouse production in Iran. As female greenhouse workers received fewer wages than male greenhouse workers, a large number of greenhouse workers are female in this area. Currently, studies have been done on the health of male greenhouse workers [17, 18], but there is a lack of knowledge for health status and occupational hazards among female greenhouse workers. In this study the general and reproductive health outcomes such as infertility, spontaneous abortion, stillbirth, preterm or postterm birth, LBW and abnormal birth between two groups of female greenhouse workers and housewives were compared.

## Methods

### Study design and participants

This comparative cross-sectional study was conducted on 308 female greenhouse workers and 337 housewives, from southern area of Iran during year of 2018. South of Kerman is divided into 18 main rural areas. At the first, these areas were arranged according to the number of greenhouses then the first 6 areas were selected. From each main rural area, five villages were selected for sampling. In each village, all female greenhouse workers aged 16–45 years with at least one-year work experience in the greenhouse and at least one-year of marriage were selected using the census method. In the control group, female were selected among housewives with lack of occupation outside of home after residence, age and marriage year adjustment. Exclusion criteria were having *underlying disease and using medicines affecting pregnancy outcomes, X-ray exposures and tobacco smoking during pregnancy. In order* to control the selection bias, the control group was selected from the nearest village to the greenhouse. Additionally, the data were collected using gender-matched interviewers to reduce the information bias.

### Data collection

Data were collected using a research-made questionnaire was developed for this study including personal, obstetrical and occupational information. Clinical assessment such as BMI, BP, PR and RR were also measured. Blood indices were measured using a blood test.

#### A- obstetrical information questionnaire

The questionnaire is including two parts; first, personal information including age, education, husband’s job and the number of children and gender. Second, obstetrical history including gravidity, spontaneous abortion rate, number of stillbirths, history of infertility and its type, abnormal infant history, preterm or postterm birth history and LBW. Infertility was divided into two primary and secondary subtypes. The primary infertility is defined as women who do not have experienced a pregnancy without using of contraception after 1 year of intercourse and secondary infertility refers to the inability of women for pregnancy after experience at least one pregnancy in the past [19]. Spontaneous abortion was considered as the ending of pregnancy before 20 weeks of gestation and stillbirth as death occurring after the 20th week of gestation [20]. Also, a newborn delivered before 37 weeks of gestation was considered as a preterm birth and a neonate delivered after 42 weeks of gestation was considered as a postterm labor [12].

#### B- occupational status questionnaire (for greenhouse workers)

The questionnaire is included occupational information such as the number of years worked and daily working hours in the greenhouse, pesticide exposure time during previous pregnancies, pesticide spray times, distance between home and greenhouse and the use of personal protective equipment while at work.

The primary questionnaire was modified by six experts in the field of reproductive health and agriculture. In both groups, data were collected in the place where they were residents, at the greenhouse for those who were worker and home for the control group. After describing the purpose of the study and obtaining written consent from the individuals, the interview was conducted in approximately 20 min.

#### C- clinical measurements (BMI, PR, RR, and BP and blood index)

The weight of participants was measured using a digital scale Zyklusmed (Zyklusmed, Hamburg, Germany) with a precision of 100 g with light clothes and no shoes. To measure height, a wall strip meter was used with a precision of 0.1 cm and without shoe. Then, BMI was calculated using each individual’s weight in kilograms and height (m^2^) based on the BMI formula (kg/m^2^). The BP, PR and RR RR were measured by sitting position after a rest of 15 min. Systolic and diastolic BP was measured using a digital pressure barometer in the right arm. The number of PR and RR was calculated in one minute. Approximately 2 ml of blood were collected from each participant and analyzed in hematology laboratory for CBC Diff test at Imam Khomeini Hospital which is affiliated to Jiroft University of Medical Sciences. All measurements were conducted by expert colleagues and all laboratory tests were free for participants. The contact details were taken from all participants involved in this study, and they were notified of the results of the tests that needed to be followed up.

### Data analysis

Data was checked for normality using the Kumologov-Smirnov test. Since data doesn’t have a normal distribution, non-parametric tests were used for further analysis. Mann-Whitney test was used for assessment variables such as age, number of children by gender, number of previous pregnancies, spontaneous abortion and clinical measurements between two groups. Chi-square test and Fisher’s exact test were used to compare the statistically significant difference in the educational status, the number of children, husband’s job, infertility, LBW, abnormal infant birth, pre-term and postterm births and stillbirth between two groups. In each comparison, the preferred test was Chi-square test. If the chi-square test assumptions were not met, only 20% of the expected cell frequencies should less than 5 and no expected cell frequencies should 0, we then used Fisher’s exact test instead. The *p*-values ≤0.05 is regarded as statistically significant. The collected data were analyzed using SPSS software version 16 (SPSS Inc., Chicago, IL, USA, 2007).

### Ethical considerations

The ethical protocol was approved by the Research Ethics Committee of Jiroft University of Medical Sciences (Approval number: IR.JMU.REC.1397.1). Participation in the study was voluntary and the purpose of the study was explained to all participants. The written informed consent was received from all the participants and greenhouse owners.

## Results

*Range of age in participants was* 16–49 years old. The mean working experience and mean daily working time of greenhouse worker were 7.91 ± 5.56 years and 6.94 ± 1.99 h, respectively.

Additionally, husband’s occupation of female greenhouse workers and control group were farmers and the percentages are significantly (*p* < 0.001) 88 and 49%, respectively.

The majority of female greenhouse workers (31.2%) and housewives (33.5%) had two children indicating a statistically significant difference between two groups (*p* = 0.002). In addition, the average number of female live birth in the greenhouse workers was significantly higher than the control group (*p* = 0.014). Some demographic characteristics of participants are presented in Table 1.

**Table 1:** The comparison of two groups of female greenhouse workers and housewives in terms of demographic variables

Variables		Greenhouse workers		Housewives		P
		Mean ± SD	N (%)	Mean ± SD	N (%)	
**Age**		33.39 ± 7.35		33.77 ± 7.51		0.521
**Education level**	≥ 12 grade		280 (91.8)		295 (89.7)	0.233
	< 12 grade		25 (8.2)		34 (10.3)	
**Number of children**	Non		23 (7.5)		8 (2.4)	0.002
	1		44 (14.3)		78 (23.1)	
	2		96 (31.2)		113 (33.5)	
	3		81 (26.3)		83 (24.6)	
	4 ≤		64 (20.8)		55 (16.3)	
**Number of children by gender**	Boy	1.29 ± 1.09		1.33 ± 1.05		0.614
	Girl	1.31 ± 1.15		1.10 ± 0.96		0.014
**Husband’s occupation**	Farmer		271 (88)		149 (44.2)	< 0.001
	self-employed		35 (11.4)		165 (49)	
	Employee		2 (0.6)		23 (6.8)	

The average number of pregnancies in greenhouse workers was significantly higher than the control group (*p* = 0.004). Forty-five percent of greenhouse workers had worked in the greenhouse during their previous pregnancies and were exposed to various types of pesticides with an average of 6.83 ± 2.54 months. The average number of pesticides sprays was 3.852 ± 6.15 times per month, and the average distance from home to greenhouse was 1.94 ± 0.91 km. Among greenhouse workers, only 44.3% (*n* = 136) were used personal protective equipment while working. The rate of infertility among greenhouse workers and control group was 14.8 and 7.5% respectively and *showed statistically significant difference* among the two study groups using Fisher’s exact test (*p* = 0.003).

The number of LBW neonates was significantly higher in greenhouse workers in comparison with controls (*p* < 0.001). Additionally 8.3% of neonates of greenhouse workers had a kind of abnormality (such as mental retardation, paralysis, *deafness, congenital heart defects, macrocephaly, blindness and* cleft lip and *palate*), which was statistically significant (*p* = 0.012). Moreover, the number of both pre-term and postterm births among greenhouse workers were higher than the control group, while only the preterm births demonstrated a significant difference (*p* = 0.035). The mean number of spontaneous abortions in greenhouse workers was higher than the control group, which was significant using Mann-Whitney test (*p* = 0.019) (Table 2).

**Table 2:** The comparison of two groups of female greenhouse worker and housewives in terms of reproductive outcomes status

Variables		Greenhouse worker		Housewives		P
		Mean ± SD	N (%)	Mean ± SD	N (%)	
**Number of previous pregnancies**		3.18 ± 1.98		2.79 ± 1.40		0.004
**Number of spontaneous abortion**^b^		0.26 ± 0.56			0.18 ± 0.50	0.019
**Infertility**	yes		45 (14.8)		25 (7.5)	0.003
	no		263 (85.4)		312 (92.5)	
**LBW**^a**,**b^	yes		32 (11.6)		10 (3.2)	< 0.001
	no		244 (88.4)		305 (96.8)	
**Abnormal births**^b^	yes		23 (8.3)		9 (2.9)	0.012
	no		253 (91.7)		306 (97.1)	
***Preterm birth***^b^	yes		33 (11.9)		19 (6.1)	0.035
	no		243(88.1)		296 (93.9)	
**Postterm birth**^b^	yes		6 (2.1)		5 (1.6)	0.509
	no		270 (97.9)		310 (98.4)	
**Stillbirth**^b^	yes		9 (3.3)		7(2.2)	0.453
	No		267 (96.7)		308 (97.8)	

^a^Low Birth Weight

^b^Regardless of primary infertility in the two groups

According to the results of Table 3, the mean of BMI in the control group was 25.91 ± 5.62, while it was 24.47 ± 5.61 among the greenhouse workers group and the difference was statistically significant based on the results of the Mann-Whitney test (*p* = 0.001). Although the mean of RR in the greenhouse workers was normal (17.03 ± 4.31), it was higher than that of women in the control group 15.47 ± 1.69 (*p* < 0.001). Blood indices measurement showed that although both groups were in the normal range, but the average number of WBC in greenhouse workers was significantly higher than the control group while mean of Hb, HCT, MCV, and MCHC of greenhouse workers were lower in comparison to control group (*p* ≤ 0.05) (Table 3).

**Table 3:** The comparison of two groups of female greenhouse worker and housewives in terms of clinical measurements

Variables		Greenhouse worker	Housewives	P
		Mean ± SD	Mean ± SD	
**BMI**^**a**^		24.47 ± 5.61	25.91 ± 5.62	0.001
**BMI**^**a**^		24.47 ± 5.61	25.91 ± 5.62	0.001
**BP**^**b**^	**Systolic**	11.44 ± 1.47	11.33 ± 1.48	0.345
	**Diastolic**	7.04 ± 1.31	7.31 ± 3.71	0.213
***PR***^**c**^		80.11 ± 10.86	79 ± 8.46	0.153
**RR**^**d**^		17.03 ± 4.31	15.47 ± 1.69	< 0.001
**RBC**^**e**^		4.85 ± 0.56	4.79 ± 0.51	0.221
**WBC**^**f**^		7.22 ± 1.82	6.80 ± 1.90	0.001
**Hb**^**g**^		12.75 ± 2.08	13.03 ± 1.40	0.05
**HCT**^**h**^		39 ± 4.32	40.22 ± 3.71	< 0.001
***MCV***^**i**^		80.28 ± 9.32	83.96 ± 6.79	< 0.001
***MCHC***^j^		26.23 ± 3.70	27.08 ± 3.04	0.002
**PLT**^k^		249.33 ± 57.40	247.25 ± 59.60	0.661

^a^Body Mass Index

^b^Blood Pressure

^c^Pulse Rate

^d^Respiratory Rate

^e^Red Blood Cells

^f^Withe Blood Cells

^g^Hemoglobin

^h^Hematocrit

^i^Mean Corpuscular Volume

^j^Mean Corpuscular Hemoglobin Concentration

^k^Platelets

## Discussion

In this study, the reproductive health outcomes among two groups of female greenhouse workers and housewives were compared. According to the results, spontaneous abortion, infertility, female infant birth, abnormal births, LBW and pre-term birth were significantly higher in the greenhouse workers. Clinical measurements indicated an increase of RR and a decrease of BMI and hematological parameters demonstrated an increase of WBC and a decrease of the Hb, HCT, MCV, MCHC among the greenhouse workers.

In this study, infertility among greenhouse workers was higher than in comparison with control group. *Pervious research indicates that* pesticide exposure can cause infertility in the farmer women due to ovarian dysfunction or hormone problems [21]. Fuortes et al. stated that the risk of the ovary or fallopian tube problems is 4–16 times higher in women employed in agricultural-associated industries [22]. According to the results most of the children in greenhouse workers were girls. There is evidence that environmental or occupational hazards including exposure to pesticides affect the gender of fetus and resulting in increased female sex ratio in comparison with male. This mechanism is due to decreased amount of Y-bearing sperm or reducing the ability of Y-bearing sperm to reach or fertilize the oocyte, exposing to pesticides for a long period of time [23]. Here, the majority spouses of the greenhouse workers are involved in farms, which is a justification for a remarkable increase in the birth rate of the female infants of this group. On the other hand, due to more *vulnerability of male fetus* compared with female, and also higher rate of abortion among the farmers [14, 24], there is likely to be more male aborted fetuses than females.

In this study, reproductive impairments including abortion, LBW, abnormal birth and pre-term birth among the greenhouse workers were significantly higher compared with housewives. Our results are consistent with previous reports showing the adverse effects of pesticide exposure on fertility [25–27]. Arbuckle et al. showed that different pesticide chemical compositions affect the time of abortion in the form of early or late spontaneous abortion [28]. Ling et al. reported that the number of preterm births is higher in women exposed to pesticides during first and second pregnancy trimesters [12]. Dabrowski et al. explained that pesticide exposure during pregnancy is associated with an elevated risk of LBW [29]. To reduce adverse outcomes, it is needed for health organizations to monitor the extent and way of pesticide use by greenhouse workers. Given that *inappropriate use* of *personal protective equipment* in greenhouse workers during the spraying of pesticides, it is necessary to provide educational programs to increase their knowledge about the adverse effects of pesticide exposure on fertility, especially in the first trimester.

In the present study, although the BMI of the greenhouse workers was normal, it was significantly lower than the control group. *This result is different from previous studies* that showed that pesticide exposure can increase weight and BMI [30, 31]. *This may be because of other factors* such as geographical, socioeconomic and nutritional culture and physical activity can affect BMI. Therefore, in order to determine the actual effects of pesticide exposure on BMI, longitudinal studies with careful examination of endocrine hormones are required through clinical trials.

The average number of RR among female greenhouse workers was higher than the control group. Many studies indicate that pesticides can effect on the lung and respiratory system resulting in increased RR, short respiration, wheezing, sore throat, impaired pulmonary function and even lung cancer [32, 33]. Therefore the annual screening of agricultural workers is necessary to identify early disorders.

In this study, blood indices assessment indicated an increase in WBC and a decrease in Hb, HCT, MCV, and MCHC in female greenhouse workers. These are in agreement with Manyilizu et al. results on Tanzanian women with more than 5 years of exposure to pesticides. They explained that the mean of MCV and HCT among exposed group was significantly lower than the control group, while the MCHC and platelets were higher in comparison to control group. They stated that prolonged exposure to pesticides affects biochemical reactions, therefore precautionary measures should be prioritized [34]. Also, the results of different studies demonstrated that increased WBC result in various types of cancers such as leukemia, neuroma and lymphoma among individuals working in the agricultural sector [35, 36]. In summary, although the blood indices are still within normal range, the significant changes of blood markers are likely due to the acute effects of exposure to various types of pesticides. This may affect other tissues of the body or develop some chronic diseases and cancers in the long time. Therefore, consecutive examination of blood biomarkers during the years of employment should be included in order to determine the health checkups of farmers.

The first limitation of this study is using cross-sectional research method that does not reflect the casual relationship. Therefore, it is suggested to consider the effects of exposure and clinical measurements using cohort and longitudinal studies. The second limitation is to measure the amount of pesticides and the condition of the use of protective equipment which has been self-reported by greenhouse workers. The final limitation of current study is the lack of evaluation of different type of pesticides used in greenhouses. The most common pesticides which have been used in the studied greenhouses including fungicide and insecticide pesticides such as Mancozeb, Abamectin, *Fenpyroximate, Daconil,* Ridomil, *Rovral-TS* that may have a potential effect on pregnancy outcomes and biochemical reactions in the blood.

## Conclusion

The results of this study demonstrated that greenhouse occupation is associated with some adverse effects on fertility and changing the level of some clinical measurements and hematological indices. Also, the use of protective equipment was reported undesirable among female greenhouse workers. Therefore, it is necessary for the future health promotion interventions of farmers to have more focus on this target group. This can be achieved by extensive collaboration between the health sector, women’s advocacy organizations and Ministry of Agriculture. Monitoring the amount and ways of using pesticides should be followed up by the relevant institutions. Workers need to attend training programs with the aim of increasing knowledge about the adverse effects of pesticide exposure, especially during pregnancy. Also, the use of protective equipment should be mandatory. The results of this study provide insight into the health status of female greenhouse workers, which is usually less studied. Future research could be helpful to identify other occupational risk factors affecting the health status of greenhouse workers.

## Supplementary information

**Additional file 1.** An English language version of the questionnaire developed for this study is provided as Additional file 1.

## Data Availability

The data sets used and/or analyzed during the current study are not publicly available due to confidentiality of data and subsequent research, but are available from the corresponding author on reasonable request.

## Abbreviations

BP: Blood Pressure
BMI: Body Mass Index
Hb: Hemoglobin
HCT: Hematocrit
LBW: Low Birth Weight
MCHC: Mean Corpuscular Hemoglobin Concentration
MCV: Mean Corpuscular Volume
PLT: Platelets
PR: Pulse Rate
RBC: Red Blood Cells
RR: Respiratory Rate
SPSS: Statistical Package for Social Sciences
WBC: White Blood Cells

## References

[1] Doss CR (2018). Women and agricultural productivity: reframing the issues. Dev Policy Rev.

[2] Rao N, Gazdar H, Chanchani D, Ibrahim M (2019). Women’s agricultural work and nutrition in South Asia: from pathways to a cross-disciplinary, grounded analytical framework. Food Policy.

[3] Neitzel RL, Krenz J, de Castro AB (2014). Safety and health hazard observations in Hmong farming operations. J Agromedicine.

[4] Mołocznik A (2004). Time of farmers' exposure to biological factors in agricultural working environment. Ann Agric Environ Med.

[5] Xiang J, Bi P, Pisaniello D, Hansen A (2013). Health impacts of workplace heat exposure: an epidemiological review. Ind Health.

[6] Torske MO, Hilt B, Glasscock D, Lundqvist P, Krokstad S (2016). Anxiety and depression symptoms among farmers: the HUNT study, Norway. J Agromedicine.

[7] Corrao CR, Mazzotta A, La Torre G, De Giusti M (2012). Biological risk and occupational health. Ind Health.

[8] Damalas CA, Koutroubas SD (2016). Farmers' exposure to pesticides: toxicity types and ways of prevention. Toxics.

[9] Nicolopoulou-Stamati P, Maipas S, Kotampasi C, Stamatis P, Hens L (2016). Chemical pesticides and human health: the urgent need for a new concept in agriculture. Front Public Health.

[10] Mrema EJ, Ngowi AV, Kishinhi SS, Mamuya SH (2017). Pesticide exposure and health problems among female horticulture workers in Tanzania. Environ Health Insights.

[11] Figà-Talamanca I (2006). Occupational risk factors and reproductive health of women. Occup Med (Lond).

[12] Ling C, Liew Z, von Ehrenstein OS (2018). Prenatal exposure to ambient pesticides and preterm birth and term low birthweight in agricultural regions of California. Toxics.

[13] Ibrahim KS, Amer NM, El Tahlawy EM, Abd Allah HM (2011). Reproductive outcome, hormone levels and liver enzymes in agricultural female workers. J Adv Res.

[14] Barrón Cuenca J, Tirado N, Vikström M, Lindh C, Steinus U, Leander K. Pesticide exposure among Bolivian farmers: associations between worker protection and exposure biomarkers. J Expo Sci Environ Epidemiol. 2019. 10.1038/s41370-019-0128-3. 10.1038/s41370-019-0128-3 PMC8608618 30787424

[15] Illing HP (1997). Is working in greenhouses healthy? Evidence concerning the toxic risks that might affect greenhouse workers. Occup Med (Lond).

[16] Ribeiro MG, Colasso CG, Monteiro PP, Pedreira Filho WR, Yonamine M (2012). Occupational safety and health practices among flower greenhouses workers from alto Tietê region (Brazil). Sci Total Environ.

[17] Lwin TZ, Than AA, Min AZ, Robson MG, Siriwong W (2018). Effects of pesticide exposure on reproductivity of male groundnut farmers in Kyauk Kan village, Nyaung-U, Mandalay region, Myanmar. Risk Manag Healthc Policy.

[18] Mehrpour O, Karrari P, Zamani N, Tsatsakis AM, Abdollahi M (2014). Occupational exposure to pesticides and consequences on male semen and fertility: a review. Toxicol Lett.

[19] Zegers-Hochschild F, Adamson GD, Dyer S, Racowsky C, de Mouzon J, Sokol R (2017). The international glossary on infertility and fertility care, 2017. Fertil Steril.

[20] Badell ML, Meaney-Delman D, Tuuli MG (2015). Risks associated with smallpox vaccination in pregnancy: a systematic review and meta-analysis. Obstet Gynecol.

[21] Bretveld RW, Thomas CM, Scheepers PT, Zielhuis GA, Roeleveld N (2006). Pesticide exposure: the hormonal function of the female reproductive system disrupted?. Reprod Biol Endocrinol.

[22] Fuortes L, Clark MK, Kirchner HL, Smith EM (1997). Association between female infertility and agricultural work history. Am J Ind Me.

[23] Terrell ML, Hartnett KP, Marcus M (2011). Can environmental or occupational hazards alter the sex ratio at birth? A systematic review. Emerg Health Threats J.

[24] Mizuno R (2000). The male/female ratio of fetal deaths and births in Japan. Lancet.

[25] Boccolini Pde M, Boccolini CS, Meyer A, Chrisman Jde R, Guimarães RM, Veríssimo G (2013). Pesticide exposure and low birth weight prevalence in Brazil. Int J Hyg Environ Health.

[26] Morris A (2018). Reproductive endocrinology: exposure to pesticide residues linked to adverse pregnancy outcomes. Nat Rev Endocrinol.

[27] Chiu YH, Williams PL, Gillman MW (2017). Association between pesticide residue intake from consumption of fruits and vegetables and pregnancy outcomes among women undergoing infertility treatment with assisted reproductive technology. JAMA Intern Med.

[28] Arbuckle TE, Lin Z, Mery LS (2001). An exploratory analysis of the effect of pesticide exposure on the risk of spontaneous abortion in an Ontario farm population. Environ Health Perspect.

[29] Dabrowski S, Hanke W, Polańska K, Makowiec-Dabrowska T, Sobala W (2003). Pesticide exposure and birthweight: an epidemiological study in Central Poland. Int J Occup Med Environ Health.

[30] LaVerda N, Goldsmith DF, Alavanja MC, Hunting KL (2015). Pesticide exposures and body mass index (BMI) of pesticide applicators from the agricultural health study. J Toxicol Environ Health A.

[31] Raafat N, Abass MA, Salem HM (2012). Malathion exposure and insulin resistance among a group of farmers in Al-Sharkia governorate. Clin Biochem.

[32] Hulse EJ, Davies JO, Simpson AJ, Sciuto AM, Eddleston M (2014). Respiratory complications of organophosphorus nerve agent and insecticide poisoning. Implications for respiratory and critical care. Am J Respir Crit Care Med.

[33] Ye M, Beach J, Martin JW, Senthilselvan A (2013). Occupational pesticide exposures and respiratory health. Int J Environ Res Public Health.

[34] Manyilizu WB, Mdegela RH, Kazwala R (2016). Association of long-term pesticide exposure and biologic parameters in female farm workers in Tanzania: a cross sectional study. Toxics.

[35] Merhi M, Raynal H, Cahuzac E, Vinson F, Cravedi JP, Gamet-Payrastre L (2007). Occupational exposure to pesticides and risk of hematopoietic cancers: meta-analysis of case-control studies. Cancer Causes Control.

[36] Del Prado-Lu JL (2007). Pesticide exposure, risk factors and health problems among cutflower farmers: a cross sectional study. J Occup Med Toxicol.

