# Supplementary material for: General and reproductive health outcomes among female greenhouse workers: a comparative study
Source: BMC Womens Health. 2020 May 13;20:103. doi: 10.1186/s12905-020-00966-y (PMC7222304; doi:10.1186/s12905-020-00966-y)
Supplement: Supplementary file 1 — Additional file 1. An English language version of the questionnaire developed for this study is provided as Additional file 1. [file 12905_2020_966_MOESM1_ESM.pdf]

“Health status and reproductive outcomes questionnaire”

ID number: _______ Group: _______

Section A: Demographic and Obstetrical information

1. What is your age: _______
2. What is your educational status: _______
3. What is your husband's occupation? _______
4. How many children do you have? _______Number of girls: _____Number of boys: _____
5. How many previous pregnancies do you have? _______
6. How many spontaneous abortions do you have? _______
7. Do you have a history of stillbirths in previous pregnancies? Yes☐ No☐
8. Do you have a history of abnormal birth in previous pregnancies? Yes☐ No☐

If yes, please state the type of abnormality: _______

9. Do you have a history of preterm birth in previous pregnancies? Yes☐ No☐
10. Do you have a history of post term birth in previous pregnancies? Yes☐ No☐
11. Do you have a history of infant birth with low weight (<2500gr) in previous pregnancies?

Yes☐ No☐

12. Do you have regular menstrual periods? Yes☐ No☐
13. Did you have a history of infertility? Yes☐ No☐

If yes, what kind of infertility do you have? Primary infertility ☐ Secondary infertility ☐

Section B: Occupational Status (only for greenhouse workers)

1. How long have you been working in the greenhouse? _______ years _______ months
2. How many hours a day do you work in the greenhouse? _______ hours
3. Have you been exposed to pesticides during previous pregnancies? Yes□ No□
   If yes, state the duration of exposure in pregnancy: _______ months

4. What is the average number of pesticide sprays per month? _______

5. What is the approximate distance of your place of residence from the greenhouse? _______ (km)

6. Do you use personal protective equipment while working in the greenhouse? Yes☐ No☐

Section C: Clinical measurements:

1. Weight: _______(kg)
2. Height: _______ (cm)
3. Body mass index: _______ kg/m^2
4. Systolic blood pressure: _______ (mmHg)
5. Diastolic blood pressure: _______(mmHg)
6. Number of breaths: _______
7. Number of pulse: _______
8. WBC: _______
9. RBC: _______
10. Hb: _______
11. HCT: _______
12. MCV: _______
13. MCHC: _______
14. PLT: _______
